# Maryland Clinical Society

**Published:** 1895-01

**Authors:** 


### Society Reports.

### MARYLAND CLINICAL SOCIETY.

### Stated Meeting held December 7th.

Dr. Rohe read a paper entitled "Clinical Observations upon the Relation of Somatic Diseases to Mental Derangement." This was followed by a paper from Dr. Preston on the "Etiology and the Pathology of Hysteria."

Dr. Wilmer Brinton read a paper on the "Induction of Labor in Nephritis," with report of cases:

I have been induced to bring the subject of the Induction of Labor in Nephritis to your notice by the reading of a paper on "The Significance of Albuminuric Retinitis in Pregnancy," written by Dr. R. L. Randolph, of this city. Dr. Randolph reports five cases of albuminuric retinitis occurring in pregnant women whom he has seen during the past two years, in which cases he decided by opthalmoscopic examination whether it was the proper treatment or not to induce labor for the purpose of saving the eyes and perhaps the life of the woman. In cases related, not only were the eyes saved where labor was induced, but in the cases where he advised the continuation of pregnancy, the women escaped eclampsia. Judging from the first case reported by Dr. Randolph, there must be some difference of opinion even among oculists as to when premature labor should be induced, for the report of this case which I shall now read will show that the first oculist consulted, advised a different method of procedure from that recommended by Dr. Randolph.

*Case*: Mrs. M., 31 years old, three children living, and up to the fourth month of her third pregnancy had enjoyed good health. In the early part of the fifth month she began to have violent headaches, which could only be relieved by strong anodynes. They persisted for two weeks, when she noticed that her sight was growing dim. It continued to grow worse until she was practically blind in one eye, and the sight in the other but little better. At this time an oculist was called in, who pronounced it albuminuric retinitis and found the urine rich in albumen and some casts present. The induction of labor was advised, performed, and a dead child born. The woman had convulsions, but recovered, with complete restoration of sight. One year later she again conceived, and in the fourth month was attacked with similar headaches. Fearing that her sight would again become bad, she consulted an oculist again, who advised that if she waited for normal labor, she would lose her sight and probably her life. Dr. Kelly was sent for to induce labor, but referred the case first to me. I found the vision twenty-fifteenths in both eyes, and a low grade of hypertropic astigmatism. I found absolutely nothing to denote progressive disease in the fundus. The question was whether or not to induce premature labor. There was a faint trace of albumen in the urine, but no casts. I concluded that the evidence did not justify the operation. My advice was followed and the patient sent home, to give birth a few months later to a boy."

The conclusions of this interesting paper were as follows:

1st. Visual disturbances occurring in the first six months of pregnancy, and especially when associated with violent headaches, frequently mean albuminuric retinitis, and if this condition is found, to *save sight*, pregnancy should be at once terminated. 2d. Visual disturbances showing themselves in the last seven weeks of pregnancy, while indicating the same retinal lesions are of less gravid import in so far as sight is concerned, and unless they are very pronounced and associated with widespread opthalmoscopic changes, should not in themselves, call for the induction of labor. 3d. The occurrence of renal retinitis in one pregnancy does not mean that the woman will be likewise affected in a subsequent one. And even though headache be present, and albumen found, so long as the fundi are free from signs of existing retinitis, the question of sight will not be considered.

The very grave prognosis in cases of eclampsia occurring in the pregnant woman, the woman in labor, or the parturient, makes the question of nephritis a very interesting one to the obstetrician. Experience and statistics prove that women who have chronic nephritis, conceive and carry their children to full term without having convulsions. Indeed, it seems that if they do not abort, they are less liable to eclampsia than women who for the first time develop kidney disease during pregnancy. Cases of nephritis occurring in the pregnant woman, whether chronic or acute in character, must make the physician in charge, anxious about the outcome of the case, for the rates of mortality vary from twenty-five to forty per cent. for the mother and from fifty to seventy-five per cent. for the child when we have eclampsia occurring during pregnancy, or before the completion of pregnancy. The question comes to us for de. cision whether we shall follow conservative treatment which at best will only ward off impending danger, or whether it is best to place the patient at once in a position of comparative safety by the induction of premature labor. Dr. Lusk says: "The weight of authority seems to me favorable to procrastination—the interruption of pregnancy being regarded as an extreme measure justifiable only in case of utmost peril. But my own convictions are clear that so soon as grave cerebral symptoms develop, the period of folded hands has passed."

The four cases I shall report have come under my notice during the past eighteen months, and while in only two cases was premature labor induced previous to convulsive movements, yet in the other two, although only seen first when in convulsions, premature labor was induced, as they were not at full time.

*Case* 1. Mrs. R., mother of nine children, and between seven and eight months advanced in her tenth pregnancy. Her physician had watched her closely for some weeks and made diagnosis of nephritis. He found albumen and casts in the urine, specific gravity 1010. Eye-sight very much impaired and rapidly growing worse; headaches violent for days, and several times have had convulsive movements. At my first visit we decided upon premature labor, and under strict antiseptic precaution, I introduced a bougie at 4 P. M., on Friday afternoon. At midnight of the next day, she was delivered of a living child. During the time of the induction of labor she had to be kept under the influence of potassium bromide and chloral hydrate. For a week or two both mother and child did well, but finally, all her symptoms grew worse, she became totally blind, went into coma, and died two months after the birth of the child.

*Case* 2. Mrs. A., 40 years of age; pregnant for the ninth time and supposed to be eight months advanced. She was blind, œdematous, pulse rapid and urine full of albumen. There were very marked indications of beginning convulsions. Treatment had been infusion of digitalis, compound jalap powder, and chloral hydrate and bromide of potash. I introduced a bougie as in case 1. Hot vaginal douches were given and some eleven hours after, the mother was delivered of a living child. Some nine months after, her physician writes me that the child died within a month but that Mrs. A. recovered with good sight.

*Case* 3. A colored out-patient with a history of eleven convulsions before my assistant saw her. An examination showed pregnancy of eight months. Child living, woman aged 17. She was removed to the hospital, chloroform, bromide of potash, and chloral hydrate given to control convulsions. Bougie was introduced, but later we had to dilate with the finger. Simpson's forceps were applied and, after great traction, a dead child delivered. The mother never regained consciousness. Died four hours later, having had fifty or sixty convulsions.

*Case* 4. Mrs. V. C., in her first confinement. All during her pregnancy had been well. Had been on the street the day previous and slept well that evening. In the morning while at breakfast, she suddenly clapped her hands to her head, cried "I cannot see" and fell to the floor in violent convulsion. Within thirty minutes she had six more. Chloroform was given during convulsions and chloral every hour during the intervals, when the patient had intelligence enough to swallow when told to do so. With the assistance of Dr. Watson, dilatation was made by the finger, Simpson's forceps applied, and a living child delivered. The woman had in the next thirty-six hours, about ten severe convulsions, and was practically unconscious for forty-eight hours afterwards. Hypodermics of morphia of one-third of a grain were used and we saw marked results for good after each dose. She gradually grew better, but complained of bad sight and violent headaches for nearly two weeks. She has done well eversince.

In the brief report of these cases, I have only mentioned a few of the many methods of inducing premature labor, but in closing, I wish to commend the method of dilating the cervix with the finger.

Dr. Michael: This question calls always for quick action, and delay is dangerous. I wish to say a word about the diagnosis. It is made often by the ophthalmologist. A doctor should make the examination of the kidney lesion himself and it should be so well-known to the obstetrician that he should not let the patient go to blindness. I should feel shabby if an ophthalmologist had to tell me of the existence of the disease. As to the treatment: I disapprove of Dr. Brinton's method of producing labor—that of using the bougie when a a woman is having convulsions. Rapid dilatation by the finger is the safest and best method of bringing it on, though it is a difficult and troublesome plan. When the hand is used, you run no risk of getting into the wrong place or doing any damage. The two remedies I like best are morphia and venesection. I do not know what venesection does except bring out a lot of bad blood, but it most surely produces good results. I believe that morphia and venesection are both extremely satisfactory, and that the latter has not been properly tried.

Dr. William T. Howard is a strong advocate of it. Before coming to Baltimore, he had treated seven cases by free bleeding and saved them all. The next six cases he saw here were treated different and all died. The next one was bled and got well. I believe the results are better on the average than are to be obtained in any other way.

Dr. Hiram Woods: The question of the eye-symptoms is apt to be misunderstood unless you bear in mind that there are two varieties of blindness associated with albuminuric conditions in pregnancy. One is the sudden failure of sight, such as described by Dr. Brinton where there is no retinal lesion; the other, a case of true inflammation, with white plaques and decided retinal changes. The question is whether in any of Dr. Brinton's cases there was true albuminuric retinitis. There were no opthalmoscopic examinations made and in all, he said, the blindness was sudden, and with one exception, all got well. I can recall a patient in my care who had albuminuric retinitis in her first pregnancy and her sight was reduced to a very small point. I followed her through four or five pregnancies and although nearly blind in each, her sight was always restored to the point it had been left during the first pregnancy. Four of Dr. Randolph's cases had these changes, the other did not. The first case of his which was referred to by Dr. Brinton was not properly diagnosed. With a woman in her first pregnancy with ensuing albuminuric retinitis, the question suggests itself, is premature labor in the fourth or fifth month justifiable? I should think it was, but how would that be regarded from an obstetrical point of view?

Dr. Todd: I find that in New York the custom among the physicians is to justify the operation for the saving of life, but not simply to save eye-sight.

Dr. Norment: I wish to mention two cases seen recently—One in her fourth pregnancy. In her first, she had eclampsia five or six weeks prior to labor and conservative treatment was adopted. She was delivered of a child which had evidently been dead for some time. In her second, she had eclampsia during labor, and was delivered by forceps of a living child. In her third, she had a perfectly normal pregnancy and labor. In the fourth, I was sent for and found her in eclampsia in the eighth month of pregnancy. She was very large, weighing 240 pounds. There was no evidence of the onset of labor, and the difficulties of inducing labor, the condition of the patient and the fact that she had been through the thing before successfully, led us temporarily to postpone the induction of labor. We followed Dr. Michael's plan and bled her freely. She was stone blind and I found any number of white plaques in the retina. Five weeks later she was delivered of a still-born child. There was little return of vision until after labor, but later it came up to about one-third normal.

Case 2: I found a woman eight months pregnant in eclampsia for several hours, recognizing no one, and complaining of pain in the head. I bled her freely, she became conscious at once and was altogether better. She had been perfectly blind, but soon was well enough to read the newspaper. She was afterwards delivered of a dead child. She had, I think, oremia without retinitis; I found no albumen in the urine.

Dr. Brinton: Where we have time certain methods can be used for inducing labor, but when in a hurry the use of the finger is best. We did use morphia in one case and with good results. I once reported four cases in which I had bled, and three recovered. In the next three treated in the same manner, all died.

